# Supplementary material for: Prevalence of intestinal parasitic infections and associated risk factors among patients attending Debarq Primary Hospital, northwest Ethiopia
Source: PLoS One. 2024 Mar 7;19(3):e0298767. doi: 10.1371/journal.pone.0298767 (PMC10919636; doi:10.1371/journal.pone.0298767)
Supplement: S1 Checklist — (DOCX) [file pone.0298767.s001.docx]

STROBE Statement—checklist of items that should be included in reports of observational studies

|  | Item No. | Recommendation | Page  No. | Relevant text from manuscript |
| --- | --- | --- | --- | --- |
| **Title and abstract** | 1 | (*a*) Indicate the study’s design with a commonly used term in the title or the abstract | 2 | health facility-based cross-sectional study |
|  |  | (*b*) Provide in the abstract an informative and balanced summary of what was done and what was found | 2 | Out of 422 individuals examined, 33.62% were infected with at least one intestinal parasite species |
| Introduction | | | |  |
| Background/rationale | 2 | Explain the scientific background and rationale for the investigation being reported | 3&4 | High burden country for IPIs, no enough study conducted in all age groups |
| Objectives | 3 | State specific objectives, including any prespecified hypotheses | 4 | To determine the prevalence of human intestinal parasite infections and associated risk factors |
| Methods | | | |  |
| Study design | 4 | Present key elements of study design early in the paper | 4 | health facility-based cross-sectional study |
| Setting | 5 | Describe the setting, locations, and relevant dates, including periods of recruitment, exposure, follow-up, and data collection | 4-6 | -study area and population  -study period (March-June, 2022)  Socio-demographic and stool data collection |
| Participants | 6 | (*a*) *Cohort study*—Give the eligibility criteria, and the sources and methods of selection of participants. Describe methods of follow-up  *Case-control study*—Give the eligibility criteria, and the sources and methods of case ascertainment and control selection. Give the rationale for the choice of cases and controls    *Cross-sectional study*—Give the eligibility criteria, and the sources and methods of selection of participants 4 Inclusion and exclusion criteria of study participants |  | Not applicable |
|  |  | (*b*) *Cohort study*—For matched studies, give matching criteria and number of exposed and unexposed  *Case-control study*—For matched studies, give matching criteria and the number of controls per case |  | Not applicable |
| Variables | 7 | Clearly define all outcomes, exposures, predictors, potential confounders, and effect modifiers. Give diagnostic criteria, if applicable | 4-6 -    -  - | prevalence of IPIs  - sociodemographic and clinical characteristics  - exclusion criteria  - stool type as diagnostic criteria |
| Data sources/ measurement | 8* | For each variable of interest, give sources of data and details of methods of assessment (measurement). Describe comparability of assessment methods if there is more than one group | *5&6 -* - | structured questionnaire was used to collect data on risk factors  - Stool sample collection and examination |
| Bias | 9 | Describe any efforts to address potential sources of bias | 5&6  .. | - A pre-tested structured questionnaire was used  -The stool examination was done independently by two qualified laboratory technicians  - All reagents and supplies used to collect and analyze stool samples were checked for quality |
| Study size | 10 | Explain how the study size was arrived at | 5 | The sample size was estimated using Cochran's single proportion formula: |

| Quantitative variables | 11 | Explain how quantitative variables were handled in the analyses. If applicable, describe which groupings were chosen and why | 7 | -The data was entered and analyzed using the statistical package for social sciences (SPSS) software version 25 |
| --- | --- | --- | --- | --- |
| Statistical methods | 12 | (*a*) Describe all statistical methods, including those used to control for confounding | 7 | -Both descriptive and logistic regression analysis methods were used. |
|  |  | (*b*) Describe any methods used to examine subgroups and interactions | 7 | Crude odds ratio (COR) and adjusted odds ratio (AOR) were calculated |
|  |  | (*c*) Explain how missing data were addressed | 7 | Not applicable |
|  |  | (*d*) *Cohort study*—If applicable, explain how loss to follow-up was addressed  *Case-control study*—If applicable, explain how matching of cases and controls was addressed  *Cross-sectional study*—If applicable, describe analytical methods taking account of sampling strategy |  | Not applicable |
|  |  | (*e*) Describe any sensitivity analyses | 7 | P-values≤ 0.05 were considered statistically significant. |
| Results | | | | |
| Participants | 13* | (a) Report numbers of individuals at each stage of study—eg numbers potentially eligible, examined for eligibility, confirmed eligible, included in the study, completing follow-up, and analysed | 7 | 422 individuals included |
|  |  | (b) Give reasons for non-participation at each stage |  | Not applicable |
|  |  | (c) Consider use of a flow diagram |  | Not applicable |
| Descriptive data | 14* | (a) Give characteristics of study participants (eg demographic, clinical, social) and information on exposures and potential confounders | 7 | 212 men and 210 women,54.02% were aged 25 and older. 57.35% of participants live in urban area. 40.02%) were married, 42.18% were illiterate |
|  |  | (b) Indicate number of participants with missing data for each variable of interest |  | Not applicable |
|  |  | (c) *Cohort study*—Summarise follow-up time (eg, average and total amount) |  |  |
| Outcome data | 15* | *Cohort study*—Report numbers of outcome events or summary measures over time |  |  |
|  |  | *Case-control study—*Report numbers in each exposure category, or summary measures of exposure |  |  |
|  |  | *Cross-sectional study—*Report numbers of outcome events or summary measures | *9-12* | *Type of IP*  *Prevalence of IPs* |
| Main results | 16 | (*a*) Give unadjusted estimates and, if applicable, confounder-adjusted estimates and their precision (eg, 95% confidence interval). Make clear which confounders were adjusted for and why they were included | 10&11 | Crude and adjusted odds ratios  Variables with p < 0.25 in univariable logistic regression were included in the multivariable logistic regression |
|  |  | (*b*) Report category boundaries when continuous variables were categorized | 12 | Yes/No, age categories, income level, |
|  |  | (*c*) If relevant, consider translating estimates of relative risk into absolute risk for a meaningful time period |  | Not applicable |

Continued on next page

| Other analyses | 17 | Report other analyses done—eg analyses of subgroups and interactions, and sensitivity analyses |  | Not applicable |
| --- | --- | --- | --- | --- |
| Discussion | | | | |
| Key results | 18 | Summarise key results with reference to study objectives | 13&14 | Overall prevalence  Predominant parasite types  Associated risk factors |
| Limitations | 19 | Discuss limitations of the study, taking into account sources of potential bias or imprecision. Discuss both direction and magnitude of any potential bias | 16 | As of limitation, the present study relied only on smear microscopy in the identification of the parasites, which has lower sensitivity than molecular methods to differentiate species such as *E. histolytica* and *E. dispar.* |
| Interpretation | 20 | Give a cautious overall interpretation of results considering objectives, limitations, multiplicity of analyses, results from similar studies, and other relevant evidence | 12-15 | -In agreement with previous studies,-----------  In contrary to other studies------  The variations can be justified by--- |
| Generalisability | 21 | Discuss the generalisability (external validity) of the study results | 13 | Discussion section |
| Other information | |  | | |
| Funding | 22 | Give the source of funding and the role of the funders for the present study and, if applicable, for the original study on which the present article is based |  | Not applicable |

Continued on next page

*Give information separately for cases and controls in case-control studies and, if applicable, for exposed and unexposed groups in cohort and cross-sectional studies.

**Note:** An Explanation and Elaboration article discusses each checklist item and gives methodological background and published examples of transparent reporting. The STROBE checklist is best used in conjunction with this article (freely available on the Web sites of PLoS Medicine at http://www.plosmedicine.org/, Annals of Internal Medicine at http://www.annals.org/, and Epidemiology at http://www.epidem.com/). Information on the STROBE Initiative is available at www.strobe-statement.org.
