# Supplementary material for: Prevalence of intestinal parasitic infections and associated risk factors among patients attending Debarq Primary Hospital, northwest Ethiopia
Source: PLoS One. 2024 Mar 7;19(3):e0298767. doi: 10.1371/journal.pone.0298767 (PMC10919636; doi:10.1371/journal.pone.0298767)
Supplement: Attachment — Submitted filename: Response to Reviewers.pdf [file pone.0298767.s007.pdf]

Response to the Academic Editor and Reviewers' Comments

Prevalence of human intestinal parasitic infections and associated risk factors in Debarq, northwest Ethiopia

Academic Editor

Dear Academic editor of the journal,

Thank you very much for your suggestions and concerns on our manuscript to fit with the journal requirements. As per your suggestion on the reference list of the manuscript, we have gone through it and made the following changes:

- Every reference on the list have been checked and corrected to meet with the journal's standard requirements. The changes are highlighted and indicated in the track change version of the revised manuscript.
- An additional reference (reference number 27) has been incorporated to the reference list and the citation numbers in the main text of the manuscript are corrected accordingly.

Reviewer #3

Dear Reviewer,

Thank you for your suggestions and comments. Please find a point-by-point response to your comments and suggestions below.

Introduction:

1. Line 57. Put space between T and full stop to read as *T. trichiura*
    - Corrected as suggested and indicated in line 60

Materials and methods:

Study area and population:

2. Line 80: Leave a space between longitude.The
    - Corrected by leaving a space as suggested, indicated in line 79

Inclusion and exclusion criteria:

3. Each of these criteria to have its own paragraph.
    - As we have been convinced by the suggestion from the previous reviewer, the sub-heading ‘Inclusion/Exclusion criteria is replaced with a more appropriate sub –heading for a manuscript which is ‘Recruitment of study participants’. The criteria for inclusion/exclusion are also included in this sub-heading.

Stool examination:

Direct wet / iodine mount:

4. Line 122: replace “was” with “were” ...., in approximately 2 grams of stool was emulsified..............
    - ‘’was’’ is replaced with ‘’were’’ as suggested, indicated in line 124
5. Line 127: delete comma after eggs in .... cysts, eggs, and trophozoites of motile intestinal parasites.
    - Corrected as suggested by the reviewer, indicated in line 129

Formal-ether concentration:

6. Line 137: Delete comma after eggs in ....if helminth eggs, larvae, cysts, and/or protozoan trophozoites
    - Comma after ‘cyst’ is deleted, but we found the comma placed after ‘eggs’ is appropriate as indicated in line 139.
7. ***Question:*** *Why did the authors use two methods of stool analysis without showing the results for each of the methods used? I do not see the need of including analysis whose results you do not use. Unless, they present the results for each of the methods used, they need to only pick one method.*
    - In our study, we have used ‘wet mount’ as an obligatory technique for the initial phase of intestinal parasites microscopic examination. In case of samples negative for intestinal parasites in the wet mount, we have used the formol-ether concentration technique. Particularly, to recover the eggs of parasites such as Schistosomes, which are naturally found in low concentration in stool and cannot

be detected by wet mount, the above supplementary method was useful. Hence, we have used both techniques in our study. The need for the two methods in our study is justified and highlighted in the track-change version of the revised manuscript, indicated from lines 131 to 133.

Moreover, as the study aimed to determine the magnitude of the intestinal parasites infection in the study area, not to compare the performance of the two methods, we have assumed that presenting the overall parasite distribution would be more appropriate.

Results:

Characteristics of the study participants:

8. Lines 150 and 151: Add "d" on the two words of "live") ……………While 57.35% of participants live in urban areas, 42.65% live in rural areas. The results have to be stated in a reported speech.
   - Corrected as suggested by the reviewer, indicated in lines 156 and 157.

Prevalence of intestinal parasitic infections:

9. Line 162: Add "mostly" between "and "and "in"……in participants aged 25 and older and in men (17.06%).
   - The statement is replaced with a more appropriate data presentation, as highlighted from lines 164-167.

Discussion:

10. Lines 218 to 219: Capitalise each word of the…………… world health organization (WHO) to read as …………World Health Organization"…………..
    - Corrected as suggested and that portion of the discussion is revised as indicated from lines 215-219.
11. Line 221: Replace "norm" with "normal" ………………resistant to the norm concentration of chlorine in drinking water

- Corrected in light of your suggestion as highlighted in lines 222 & 223.

12. Line 229: …………. area, such issues must be identified and addressed. Please note that this study has already identified them and I guess what is needed now is to address them. Thus delete: “must be identified and”
    - Corrected as suggested and highlighted from lines 229-231.
13. Line 233: Add “were” between residents and engaged in ……………….. and 37.5% of rural residents engaged in open defecation,
    - In light of your suggestion, the paragraph is modified in a more appropriate manner
14. Line 237: See previous comment made on Line 229. Please delete “identify and” in ………………. it is essential to identify and address sanitary facility concerns in…………..
    - As per your suggestion, the statements are rewritten in a more appropriate way
15. Line 248: Replace “are” with “is” in …………….. launching similar programs are likely to reduce the prevalence of intestinal………… because this sentence is talking about the implementation of the WASH programme.
    - The statement is rewritten to incorporate more relevant concepts
16. Line 259: Add “be” between to and associate in …………..has also been found to associate with an increased risk of intestinal………….. Further, replace “associate” with “associated”
    - Corrected as suggested with modification to the statement
17. Line 273: Replace “are” with “being” in ……………….with protozoan parasites (E. histolytica and G. lamblia) are predominantly…………
    - Corrected as suggested and highlighted in lines 273 & 274
18. Line 274: Delete “a” on “abe” in ……………… of intestinal parasites was observed to abe associated…………………….
    - Corrected as per your suggestion and highlighted in line 275 of the track change version of the revised manuscript.

Reviewer #1

Dear Reviewer,

Thank you for your suggestions and comments. Please find a point-by-point response to your comments and suggestions below.

1. Stool sample collection- Line 113-t0 155 can be rephrased. Also, in line 144, use past tense instead of present tense.
   - We understand that the comment was for lines 133 to 115 (not 155). Hence, we have tried to rephrase the statement as per your suggestion and highlighted, as indicated from lines 113-116 of the track version of the revised manuscript.
   - Unfortunately, we could not get present tens in line 144 as commented by the reviewer; hence, we were not able to correct it as suggested.
2. The conclusion can be improved by a more detailed recommendation. Health education can be changed to health promotion.
   - In agreement with your suggestion, we tried to improve the conclusion with additional recommendation points as highlighted and indicated from lines 279-282.
   - As suggested, health ‘education’ is replaced with health ‘promotion’, indicated/highlighted in line 278 of the track version of the revised manuscript.

Thank you All,

The corresponding author of the manuscript
