# Supplementary material for: Prevalence of intestinal parasitic infections and associated risk factors among patients attending Debarq Primary Hospital, northwest Ethiopia
Source: PLoS One. 2024 Mar 7;19(3):e0298767. doi: 10.1371/journal.pone.0298767 (PMC10919636; doi:10.1371/journal.pone.0298767)
Supplement: Attachment — Submitted filename: Plos one comments.docx [file pone.0298767.s002.docx]

Prevalence of human intestinal parasitic infections and associated risk factors in Debarq, northwest Ethiopia

1. Abstract- Line 30; how where the study participants recruited
2. **Introduction**
3. In paragraph one please add the African burden of IIPs
4. Line 55- was should be replaced with have
5. Line 57- T.trichiura is appearing for the first time and should be written in full.
6. Line 61-63 is a repletion of line 50-52 and should be deleted
7. Line 68-69 that statement is not true
8. The introduction can be improved, it is information about the local context what about elsewhere? What are the current WHO recommendations about prevention and control of the parasites?
9. **Methods**
10. Sub -heading inclusion criteria is not needed in manuscript writing- can sampling procedure/recruitment of study participants
11. Sub heading 100- can be replace with questionnaire survey
12. Line 101; how where the study participants recruited?
13. **Results**
14. Table three can be replaced with table 4
15. **Discussion**
16. Line 212- what about other countries with similar problems; what is their prevalence level?
17. The discussion is very scanty- most of the associated risk factors have not been discussed and a reason given why they were associated and what others have found with similar studies.
18. The discussion needs a lot of revision- all the associated risk factors needs to be discussed.
19. Addition of qualitative data would have given more insight as to why this IIPs are still a problem.
