# Supplementary material for: Prevalence of intestinal parasitic infections and associated risk factors among patients attending Debarq Primary Hospital, northwest Ethiopia
Source: PLoS One. 2024 Mar 7;19(3):e0298767. doi: 10.1371/journal.pone.0298767 (PMC10919636; doi:10.1371/journal.pone.0298767)
Supplement: Attachment — Submitted filename: PONE-D-23-15672_Annotated by Editor HMA.docx [file pone.0298767.s003.docx]

**Prevalence of intestinal parasitic infections and associated risk factors in Debarq, northwest Ethiopia**

Amir Alelign^1*^, Nigus Mulualem ^1^, Zinaye Tekeste^2^

^1^Department of Biolog, College of Natural and Computational Science, University of Gondar, Gondar, Ethiopia

^2^ Aklilu Lemma Institute of Pathobiology, Addis Ababa University, Addis Ababa, Ethiopia

^*^ Corresponding author

E-mail: [aleamiro2009@gmail.com](mailto:aleamiro2009@gmail.com) (AA)

[nigusmulu21@gmail.com](mailto:nigusmulu21@gmail.com) (NM)

[zinaye.tekeste@aau.edu.et](mailto:zinaye.tekeste@aau.edu.et) (ZT)

**Abstract**

Intestinal parasite infections (IPIs) are major public health problems in developing countries. This study was conducted to determine the prevalence and associated risk factors of IPIs at Debarq Primary Hospital in northwest Ethiopia. A health facility-based cross-sectional study was conducted from March 2022 to June 2022. Stool samples were collected from patients attending Debarq Primary Hospital and examined for intestinal parasites using direct wet-mount and formal-ether concentration techniques. Socio-demographic data were collected using a structured questionnaire. Out of 422 individuals examined, 33.62% were infected with at least one intestinal parasite species. About 12.79%, 8.53%, 7.10%, 1.65%, 0.71%, 0.24, and 0.24% of the participants were infected with Entamoeba histolytica/dispar, Giardia lamblia, Ascaris lumbricoides, hookworm, Schistosoma mansoni, Hymenolepis nana, and Trichuris trichiura, respectively. Double and triple parasite infections were 2.37% and 0.24%, respectively. Not washing hands after using the toilet (Adjusted odds ratio (AOR) =4.42, Confidence interval (CI) = 3.11–5.90), eating unwashed vegetables (AOR = 4.90, CI = 3.28–7.32), not washing hands before eating (AOR = 2.62, CI =3.27-8.73), and not having a latrine (AOR = 4.76, CI = 4.66–8.17) were significantly associated with IPIs. This study demonstrates that IPIs are common among patients attending Debarq Primary Hospital and are associated with personal hygiene and sanitation practices, necessitating prompt control and preventative measures at both the individual and communal levels.

**Key words/phrases:** Intestinal parasites, prevalence, risk factors, Debarq, Ethiopia

**Introduction**

Intestinal parasite infections (IPIs) continued to be one of the world's major public health problems [1, 2]. An estimated 3.5 billion people worldwide are infected with intestinal parasites [3] . Trichuriasis, amoebiasis, ascariasis, and hookworm are the most common intestinal parasitic diseases globally [4, 5]. Compared to developed countries, developing countries have a higher burden of IPIs [5, 6]. The high burden of IPIs in developing countries has been linked to a rising population, poor environmental sanitation, inadequate toilet facilities, a lack of access to clean water, poverty, and climate change [7, 8].

In Ethiopia, studies examined the prevalence of IPIs and associated risk factors, and the prevalence was shown to vary by region [9, 10]. For instance, infection with *A. lumbricoides* was more common in the highlands of Ethiopia (29%), the temperate regions (35%), and the lowlands (38%). While *T.trichiura* infection showed comparable prevalences in all altitudinal regions (13% on average), hookworm infection had the highest prevalence in the lowlands (24%) followed by temperate (15%) and highlands (7%) [9, 10]. However, the prevalence of IPIs in some areas of Ethiopia is unknown.

Several factors contributed to Ethiopia's prevalence of intestinal parasitic infections, including low living standards, inadequacy or lack of clean water supply, low socioeconomic status, poor environmental sanitation, and unsafe human waste disposal systems [11]. It has also been reported that Ethiopia has one of the world's lowest-quality drinking water supplies and latrine coverage, which is likely to contribute to the country's high prevalence of IPIs [12]. However, in many parts of Ethiopia, the role of the above-mentioned other factors in increasing the prevalence of IPIs is unknown [13, 14].

There has been no study of the prevalence of IPIs and associated risk factors in various parts of Ethiopia [13, 14]. The majority of previous studies carried out in Ethiopia also focused on under-fives and school children [15-18]. However, determining the prevalence of intestinal parasites in all age groups aids in the development of effective preventative plans that address all groups of people in the country [19, 20]. Thus, this study was conducted to determine the prevalence of intestinal parasite infections and associated risk factors among patients attending Debarq Primary Hospital in northwest Ethiopia.

**Materials and methods**

**Study area and population**

A health facility-based cross-sectional study was conducted from March to June 2022. The study was carried out at Debarq Primary Hospital in northwest Ethiopia. Debarq town is located 282 kilometers from Bahir Dar, the capital city of the Amhara region. It is 2850 meters above sea level and lies between 13°08'N latitude and 37°54′E longitude.The town has a total population of 59,920 (30615 males and 29,305 females) [21]. The inhabitants' livelihoods are built on a sustainable mixed farming system. Except for a few public health facilities, including Debarq Primary Hospital, Debark does not have adequate access to health care [21].

**Inclusion and exclusion criteria**

Individuals who visited the Debarq primary hospital seeking treatment during the study period (March to June 2022) were recruited and included for the study based on the following criteria. Individuals who lived in the study area for at least one month before data collection and gave written consent to participate in the study were included in the study. Individuals who had recently taken antiprotozoal or anthelminthic drugs, as well as those who were critically ill during data collection, were excluded from the study.

**Sample size determination and sampling technique**

The sample size was estimated using single proportion formula [22].

n = Z_α/2_^2^ *p*(1-p) / d^2^, n = [(1.96)^2^ 0.5 (1-0.5)]/ (0.05)^2^= 384. Where; d is the margin of error (0.05), p is the population proportion (50%), and Zα/2 is the normal distribution's critical value at α/2 (for 95% CI, α is 0.05 and the critical value is 1.96). With a 10% non-response rate, the sample size was estimated to be 422. Study participants were selected using simple random sampling and every person who was eligible to participate in the study was interviewed based on their order of arrival

**Socio-economic and risk factor data collection**

A pre-tested structured questionnaire was used to collect data on risk factors of intestinal parasites and the study participants' socioeconomic characteristics. The socioeconomic characteristics collected from the participants included age, gender, residence, marital status, education status, occupation, and monthly income. The participants were asked questions on a range of potential risk factors, including access to toilets and clean water, wearing shoes, washing hands before eating, consuming raw meat, and consuming unclean or contaminated vegetables.

**Stool sample collection**

The participants were provided clean labeled stool collection cups and applicator sticks, and they were instructed to submit approximately 5 grams of stool. Each stool collection cup was labeled with the date of sampling as well as the name, age, and gender of the person who provided the samples. The stool samples collected from each participant were examined within 30 minutes of collection at Debark Primary Hospital's parasitology laboratory. The stool examination was done independently by two qualified laboratory technicians. All reagents and supplies used to collect and analyze stool samples were checked for quality.

**Stool examination**

**Direct wet / iodine mount**

Following a macroscopic examination of the stool samples for consistency, color, and the presence of blood, mucus, and adult intestinal helminths, a direct wet mount was prepared and examined as described elsewhere [23]. Briefly, approximately 2 grams of stool was emulsified with normal saline (0.85% sodium chloride (NaCl) solution), and then a drop of the emulsified sample was placed on a clean microscopic glass slide. An iodine mount was made on the other side of the slide. Coverslips were placed at a 45° angle to cover both wet mounts. Finally, the samples were examined under a light microscope with 10x and 40x objective lenses for larvae, cysts, eggs, and trophozoites of motile intestinal parasites.

**Formal-ether concentration**

A portion of the stool samples collected from the participants was examined using the formal-ether concentration technique as described elsewhere [23]. Briefly, around 1-2 grams of stool samples were placed in a clean 15 ml conical tube containing 7 ml of 10% formalin using a wooden applicator stick. After mixing the sample with the applicant, the suspension was filtered through a sieve (cotton gauze) into a beaker, and the filtrate was placed in the same tube. After adding 3 mL of diethyl ether to the mixture and hand-shaking it, the contents were centrifuged at 2000 rpm for 3 minutes. The sediments were used to prepare an iodine stain. Finally, the entire area under the coverslip was examined with 10x and 40x objective lenses. Specimens were considered positive if helminth eggs, larvae, cysts, and/or protozoan trophozoites were found using either wet/iodine mount or formal ether concentration techniques.

**Data analysis**

The data was entered and analyzed using the statistical package for social sciences (SPSS) software version 25 (Armonk, NY: IBM Corp.). Crude odds ratio (COR) and adjusted odds ratio (AOR) were calculated using logistic regression to examine the association between socioeconomic characteristics, risk factors for intestinal parasites, and intestinal parasite infections. Variables with p < 0.25 in univariable logistic regression were included in the multivariable logistic regression. P-values ≤ 0.05 were considered statistically significant.

**Results**

**Characteristics of the study participants**

Table 1 shows the socio-economic characteristics of the study participants. A total of 422 individuals (212 men and 210 women) participated in the study. The majority of the participants (54.02%) were aged 25 and older. While 57.35% of participants live in urban areas, 42.65% live in rural areas. The majority of participants (40.02%) were married, 42.18% were illiterate, 43.60% had a household size of 4 to 6, and 56.87% earned less than 1000 Ethiopian birr (ETB) a month.

**Table 1. General characteristics of the study participants (N = 422)**

| Variables | Categories | Number | Percentage |
| --- | --- | --- | --- |
| Sex | Male | 212 | 50.20 |
|  | Female | 210 | 49.80 |
| Age group (years) | <15 | 78 | 18.48 |
|  | 15-24 | 116 | 27.49 |
|  | ≥ 25 | 228 | 54.02 |
| Residence | Urban | 242 | 57.35 |
|  | Rural | 180 | 42.65 |
| Marital status | Single | 134 | 31.75 |
|  | Married | 169 | 40.05 |
|  | Divorced | 77 | 18.25 |
|  | Widowed | 15 | 3.55 |
| Education level | Illiterate | 178 | 42.18 |
|  | Primary school | 102 | 24.17 |
|  | Secondary school | 90 | 21.33 |
|  | Tertiary education | 52 | 12.32 |
| Occupational status | Government employer | 131 | 31.04 |
|  | Merchant | 50 | 11.85 |
|  | Farmer | 84 | 19.91 |
|  | Housewife | 66 | 15.64 |
|  | Student | 91 | 21.56 |
| Family size | ≤ 3 | 106 | 25.12 |
|  | 4-6 | 184 | 43.60 |
|  | 7-9 | 70 | 16.59 |
|  | >9 | 62 | 14.69 |
| Monthly Income (ETB) | <1000 | 240 | 56.87 |
|  | 1000-3000 | 108 | 25.59 |
|  | >3000 | 74 | 17.54 |

ETB= Ethiopian birr.

**Prevalence of intestinal parasitic infections**

The prevalence of single, double, and triple parasite infections among the study participants is shown in Table 2. Seven intestinal parasites (two protozoans and five helminths) were identified in the study participants. One or more intestinal parasites were identified in 33.64% of the study participants. Single and double parasite infections were identified in 66 (15.64%) and 6 (1.42%) of the male participants, respectively. The majority of parasitic infections (14.45%) were found in participants aged 25 and older and in men (17.06%).

**Table 2. Intestinal parasite infections among study participants by gender and age group (N = 422).**

| **Types of intestinal parasites** | **Sex** | | |  | **Age (years)** | | | | |
| --- | --- | --- | --- | --- | --- | --- | --- | --- | --- |
|  | Male no. (%) | Female no. (%) | Total no.  (%) |  | 5-14  no. (%) | 15-24  no. (%) | | ≥25  no.(%) | Total  Total number (%) |
| *E.histolytica/dispar* | 22(5.21) | 32(7.58) | 54(12.79) |  | 18(4.26) | | 11(2.60) | 25(5.92) | 54(12.79) |
| *G.lamblia* | 23(5.45) | 13(3.08) | 36(8.53) |  | 12(2.84) | | 8(1.89) | 16(3.79) | 36(8.53) |
| *A.lumbricoides* | 11(2.60) | 19(4.50) | 30(7.10) |  | 12(2.84) | | 3(0.71) | 15(3.55) | 30(7.10) |
| Hookworm | 6(1.42) | 1(0.23) | 7(1.65) |  | 1(0.23) | | 2(0.47) | 4(0.94) | 7(1.65) |
| *H.nana* | 1(0.23) | 0(0.00) | 1(0.23) |  | 1(0.23) | | 0(0.00) | 0(0.00) | 1(0.23) |
| *S.mansoni* | 3(0.71) | 0(0.00) | 3(0.71) |  | 1(0.23) | | 1(0.23) | 1(0.23) | 3(0.23) |
| Total | 66(15.64) | 65(15.40) | 131(31.04) |  | 45(10.66) | | 25(5.92) | 61(14.45) | 131(31.04) |
| Double infection (n=10) |  |  |  |  |  | |  |  |  |
| *E. histolytica & G.lamblia* | 3(0.71) | 1(0.23) | 4(0.95) |  | 1(0.23) | | 1(0.23) | 2(0.47) | 4(0.94) |
| *E. histolytica & A. lumbricoides* | 0(0.00) | 1(0.23) | 1(0.23) |  | 0(0.00) | | 1(0.23) | 0(0.00) | 1(0.23) |
| *E. histolytica & hookworm* | 1(0.23) | 0(0.00) | 1(0.23) |  | 1(0.23) | | 0(0.00) | 0(0.00) | 1(0.23) |
| *E. histolytica & S.mansoni* | 1(0.23) | 0(0.00) | 1(0.23) |  | 0(0.00) | | 1(0.23) | 0(0.00) | 1(0.23) |
| *E. histolytica & T. trichiura* | 0 | 1(0.23) | 1(0.23) |  | 0(0.00) | | 0(0.00) | 1(0.23) | 1(0.23) |
| *G. lamblia & hookworm* | 0(0.23) | 1(0.23) | 1(0.23) |  | 0(0.00) | | 0(0.00) | 1(0.23) | 1(0.23) |
| *A. lumbricoides & hookworm* | 1(0.23) | 0(0.00) | 1(0.23) |  | 0(0.00) | | 1(0.23) | 0(0.00) | 1(0.23) |
| Total | 6(1.42) | 4(0.95) | 10(2.37) |  | 2(0.47) | | 4(0.95) | 4(0.95) | 10(2.37) |
| Triple infection (n=1) |  |  |  |  |  | |  |  |  |
| *E.histolytica &G.lamblia & T.trichiura* | 0(0.00) | 1(0.23) | 1(0.23) |  | 0(0.00) | | 0(0.00) | 1(0.23) | 1(0.23) |
| Total | 0(0.00) | 1(0.23) | 1(0.23) |  | 0(0.00) | | 0(0.00) | 1(0.23) | 1 (0.23) |

The most commonly found parasite identified in the study participants was *E. histolytica/dispar*, 54 (12.79%), followed by *G. lamblia* 36 (8.53%), *A. lumbricoides* 30 (7.10%), hookworm (1.65%), *H. nana* (0.23%), and *S. mansoni* (0.71%). *E. histolytica/dispar* and *G. lamblia* caused the highest proportion of double infections (0.94%). Only 0.23% of females and individuals aged 25 and older had triple infections with *E. histolytica*, *G. lamblia*, and *T. trichiura*.

**Associations of intestinal parasitic infections with socio-economic factors**

Table 3 shows the association between socio-demographic factors and intestinal parasite infections. Participants under the age of 15 were 3.77 times more likely to be infected with intestinal parasites than those aged 15–24 (COR = 3.77, CI = 1.37-5.63). There were higher odds of intestinal parasite infection in those with a monthly income of less than 1000 ETB per month compared to those earning more than 3000 ETB per month (COR=2.74, CI = 3.4-5.73).What about those earning 1000-3000?

**Table 3. Univariate analysis for potential risk factors of intestinal parasitic infections among the participants (N = 422)**

| Variables | Categories | N (%) | IPIs |  | COR ( 95%CI) P-value |
| --- | --- | --- | --- | --- | --- |
|  |  |  | Positive (%) | Negative (%) |  |
| Sex | Male | 212 (50.20) | 72 (17.10) | 140(33.20) | 1.00 ( 0.68-1.49) 0.99 |
|  | Female | 210 (49.80) | 70 (16.60) | 140(33.20) | 1 |
| Age group  (years) | <15 | 78 (18.48) | 47 (11.10) | 31(7.30) | 3.77(1.37-5.63) 0.005* |
|  | 15-24 | 116 (27.49) | 29 (6.90) | 87(20.60) | 1 |
|  | ≥25 | 228 (54.02) | 66 (15.60) | 162(38.40) | 8.6 (4.9-5.47) 0.57 |
| Residence | Urban | 242 (57.35) | 76 (18.00) | 166(39.30) | 1 |
|  | Rural | 180 (42.65) | 84 (19.90) | 96(22.70) | 6.70 (4.49-9.98) 0.15 |
| Marital status | Married | 134(31.75) | 40(31.80) | 94(22.30) | 1 |
|  | Single | 169(40.05) | 64(15.20) | 132(31.30) | 0.80 ( 0.52-1.23) 0.31 |
|  | Divorced | 77(18.25) | 48(11.40) | 29(6.90) | 2.73 (1.03-7.19) 0.35 |
|  | Widowed | 15(3.55) | 9(2.10) | 6(1.40) | 1.25 ( 0.32-4.88) 0.75 |
| Educational | Illiterate | 178(42.18) | 86(20.40) | 92(21.80) | 4.77 (3.37-5.63) 0.52 |
|  | Primary | 102(24.17) | 35(7.30) | 67(15.90) | 3.37 (4.10-5.13) 0.34 |
|  | Secondary | 90(21.33) | 16(3.80) | 74(17.50) | 3.10 (3.35-7.16) 0.35 |
|  | Diploma and above | 52(12.32) | 5(1.20) | 47(11.10) | 1 |
| Monthly income  (ETB) | < 1000 | 240(56.87) | 158(37.40) | 82(19.40) | 2.74 ( 3.4-5.73) 0.006* |
|  | 1000-3000 | 108(25.59) | 70(16.60) | 38(9.00) | 1.61 (1.90-2.87) 0.016* |
|  | > 3000 | 74(17.54) | 22(5.2) | 52(12.3) | 1 |
| Family size | ≤3 | 106(25.12) | 28(6.60) | 78(18.50) | 1 |
|  | 4-6 | 184(43.60) | 120(28.40) | 63(14.90) | 90 ( 0.81-4.49) 0.14 |
|  | 7-9 | 70(16.59) | 46(10.90) | 24(5.70) | 1.56 (0.66-3.65) 0.31 |
|  | >9 | 62(14.69) | 35(8.30) | 27(6.40) | 1.39 ( 0.50-3.87) 0.53 |
| Occupation | Government employers | 131(31.04) | 42(9.90) | 89(21.10) | 1 |
|  | Merchants | 50(11.85) | 23(5.50) | 27(6.40) | 0.75 ( 0.32-1.77) 0.51 |
|  | Farmers | 84(19.91) | 49(11.60) | 35(8.30) | 0.59 ( 0.35-1.00) 0.05 |
|  | Housewives | 66(15.64) | 28(6.60) | 46(10.90) | 0.59 (0.23-1.55) 0.29 |
|  | Students | 91(21.56) | 8(1.90) | 83(19.70) | 0.74 (0.39-1.38) 0.34 |

1, reference value; *, statistically significant; COR, crude odds ratio; N, the total number of cases; ETB, Ethiopian Birr; IPIs, intestinal parasite infections.

**Logistic regression analysis for potential risk factorsof intestinal parasitic infections**

Table 4 shows a logistic regression analysis of factors affecting IPIs. Participants under the age of 15 were 2.37 times more likely to be infected with intestinal parasites than those aged 15–24 (AOR = 2.37, CI = 1.37–5.63). There were higher odds of intestinal parasite infection in those with a monthly income of less than 1000 ETB per month compared to those earning more than 3000 ETB per month (AOR = 2.3, CI = 3.4–5.7).

Not washing hands before food (AOR=2.43, CI= 2.72-5.37), not hand washing after using the toilet (AOR=4.42, CI=3.11-5.90), eating unclean or unwashed vegetables (AOR=4.90, CI=3.28-7.32), and a lack of a latrine (AOR=3.36, CI=4.66-8.17) were all associated with a higher odds of IPIs.

**Table 4. Multivariable logistic regression analysis for potential determinants of intestinal parasitic infections among the participants (N = 422)**

| Risk factors | Categories | N (%) | IPIS |  | AOR, (95%CI) P-value |
| --- | --- | --- | --- | --- | --- |
|  |  |  | Positive (%) | Negative (%) |  |
| Hand washing before eating | Yes | 406(96.20) | 131 (31.0) | 275(65.20) | 1 |
|  | No | 16(3.80) | 11(2.60) | 5(1.20) | 2.43(2.72-5.37) 0.04* |
|  |  |  |  |  |  |
|  |  |  |  |  |  |
| Age group | <15 | 78(18.48) | 47(11.10) | 31(7.30) | 2.37(1.37-5.63) 0.007* |
|  | 15-24 | 116(27.49) | 29(6.90) | 87(20.60) | 1 |
|  | ≥25 | 228(54.02) | 66(15.60) | 162(38.40) | 4.6(4.9-5.47) 0.571 0.056 |
|  |  |  |  |  |  |
| Monthly income (ETB) | < 1000 | 240(56.87) | 158(37.40) | 82(19.40) | 2.3(3.4-5.73) 0.04* |
|  | 1000-3000 | 108(25.59) | 70(16.60) | 38(9.00) | 1.61(1.90-2.87) 0.16 |
|  | > 3000 | 74(17.54) | 22(5.20) | 52(12.30) | 1 |
| A habit of hand washing after using the toilet | Yes | 237(56.20) | 78(18.50) | 159(37.70) | 1 |
|  | No | 185(43.80) | 121(28.70) | 64(15.20) | 4.42(3.11-5.90) < 0.001* |
| A habit of eating unwashed vegetable | Yes | 197(46.70) | 123(29.10) | 74(17.50) | 4.90(3.28-7.32) 0.003* |
|  | No | 225(53.30) | 68(16.10) | 157(37.20) | 1 |
| Presence of latrine at home | Yes | 187(44.30) | 56(13.30) | 131(31.00) | 1 |
|  | No | 235(55.70) | 149(35.30) | 86(20.40) | 3.36(4.66-8.17) 0.002* |
|  |  |  |  |  |  |
|  |  |  |  |  |  |

1 , reference value; * , statistically significant; AOR, adjusted odds ratio; N, the total number of cases; ETB, Ethiopian Birr; IPIs, intestinal parasite infections

**Discussion**

Determining the magnitude of intestinal parasite infections and identifying associated risk factors in a certain population is important for the development, implementation and evaluation of preventive and control measures. This study assessed the prevalence of human intestinal parasite infections and associated risk factors among patients attending Debark Primary Hospital in northwest Ethiopia.

The overall prevalence of intestinal parasite infections in this study (33.64%) is lower than a report from Aksum, north Ethiopia (44.6%) [24]. but higher than that found in southern Ethiopia (26.2%) [25]. Limited access to clean water, poor environmental sanitation, overpopulation, and low altitude have all been associated with an increase in the prevalence of IPIs [26]. The discrepancies in the prevalence of intestinal parasite infections between this study and those conducted in different parts of Ethiopia may be explained by variances in the aforementioned factors.

The predominant intestinal parasite identified in the present study was *E. histolytica/dispar*, (12.79%), followed by *G. lamblia* (8.53%), These parasites are also formed the most concurrently appeared mixed infections in this study, which is consistent with findings in northwest and southern Ethiopia (5, 27, 28). The higher prevalence of these two protozoan parasites might be explained by the fact that they have a feco-oral transmission route and higher reproduction and persistence capacity in the environment and the host. According to the world health organization (WHO) report, the cysts of E. histolytica are very resistant and can survive for several months in water at a wider temperature range of 0 °C to 50 °C, and are extremely resistant to the norm concentration of chlorine in drinking water [29].

In Ethiopia, access to safe and clean water is limited, particularly in rural regions. Water scarcity in the country was also associated with the country's arid climate and irregular rainfall distribution [29]. Furthermore, it has been reported that water sources in the country are heavily contaminated with human feces, agricultural products, and animal waste [30, 31]. All of the factors mentioned above that have been linked to an increased risk of intestinal parasite infections elsewhere may have contributed to the high prevalence of intestinal parasite infections in the study areas. Thus, to reduce the prevalence of intestinal parasite infections in the study area, such issues must be identified and addressed.

In Ethiopia, inadequate sanitary facilities such as toilets and latrines pose a significant concern [30, 31]. The percentage of improved toilet use (not shared) is merely 6% [32]. This has been reported to encourage open defecation, contaminating the country's water sources [30, 31, 33]. It has been reported that 8.7% of urban and 37.5% of rural residents engaged in open defecation, and 82.5% of urban and 97.5% of rural residents lacked access to adequate sanitation [34]. Such contamination encourages the spread of intestinal parasites. This may be the reason for the high prevalence of intestinal parasite infection in the study area. Thus, to reduce the prevalence of intestinal parasite infections, it is essential to identify and address sanitary facility concerns in the study area.

Many individuals in Ethiopia lack adequate knowledge of good hygiene practices such as handwashing, which can lead to the spread of intestinal parasites [35]. This may also be one of the reasons for the high prevalence of intestinal parasite infection in the study area. In 2013, the Ethiopian government started the WASH National Program, with some of its goals being reducing open defecation and enhancing public sanitation, such as hand washing and improving sanitary facilities [35, 36]. The program has had considerable results, including a 17% reduction in open defection and an improvement in hygienic habits in schools and households [30, 35, 36]. However, there are several areas in the country where such a program has received little attention [30, 35, 36]. Thus, implementing this program in areas where it has received little attention and launching similar programs are likely to reduce the prevalence of intestinal parasite infections.

In this study, the risk of intestinal parasitic infection was found to be higher in participants aged 15 or younger. Intestinal parasite infections affect people of all ages; however, school-aged children (6–15 years) are the most affected [14, 37-39]. Children are more vulnerable to intestinal parasite infection due to their low immunity and frequent play in fecal-contaminated soil, which increases their risks of infection [40, 41]. They are also more likely to be exposed to protozoan and helminthic infections than other age groups due to poor hygiene [42]. The aforementioned reasons could explain the increased risk of intestinal parasite infections in children observed in this and previous studies.

In this study, a low income (less than ETB 1,000) was linked to an increased risk of intestinal parasite infection, which is consistent with the findings of other similar studies [43-46]. Income has also been found to associate with an increased risk of intestinal parasite infections in other regions of Ethiopia [18, 47-49]. People with higher incomes have higher access to sanitation supplies such as soap, toilets, and other facilities, which reduces their chances of contracting intestinal parasites [50-52].

The finding in this study that IPIs are associated with poor personal hygiene, such as not washing hands before eating and after using the bathroom, is consistent with findings in other parts of the country and elsewhere [53-56]. This could be because poor personal hygiene, such as not washing hands before eating and after using the bathroom, has been linked to the spread of intestinal parasite infections [57]. Moreover, this study found that eating unwashed vegetables is associated with an increased risk of intestinal parasite infections. Fruits and vegetables can be contaminated with the infective stages of intestinal parasites, and eating them without washing increases the risk of infection [58].

?**Conclusions**

The prevalence of human intestinal parasites in Debarq, northwest Ethiopia was found to be relatively high with protozoan parasites (*E. histolytica and G. lamblia*) are predominantly identified. This considerabily high prevalence of intestinal parasites was observed to abe associated with age, inadequate sanitary facilities and practice, low income level and poor personal hygiene. It is therefore, important to address at risk groups such as children, promote hand washing practices before food eating and after defication, and the use of latrines. As of limitation, the present study relaid only on smear microscopy in the identification of the parasites, which has lower sensitivity than molecular methods to differentiate species such as *E. histolytica* and *E. dispar.*

**Acknowledgements**

We would like to thank all data collectors, study participants and local health office authorities in the study area for their willingness and cooperation during data collection.

**References**

1. Sitotaw B, Mekuriaw H, Damtie D. Prevalence of intestinal parasitic infections and associated risk factors among Jawi primary school children, Jawi town, north-west Ethiopia. BMC Infect Dis. 2019;19(1):341.
2. Belete YA, Kassa TY, Baye MF. Prevalence of intestinal parasite infections and associated risk factors among patients of Jimma health center requested for stool examination, Jimma, Ethiopia. PLoS One. 2021;16(2):e0247063.
3. World Health Organization. 2020. https://www.who.int/news-room/fact-sheets/detail/soil-transmitted-helminth-infections. Global distribution and prevalence of soiltransmitted helminth infections. Accessed on 9 January 2022.
4. Hailu T, Abera B, Mulu W, Kassa S, Genanew A, Amor A. Prevalence and factors associated with intestinal parasitic infections among pregnant women in West Gojjam zone, northwest Ethiopia. J Parasitol Res. 2020;2020.
5. Eyayu T, Kiros T, Workineh L, Sema M, Damtie S, Hailemichael W, et al. Prevalence of intestinal parasitic infections and associated factors among patients attending at Sanja Primary Hospital, Northwest Ethiopia: An institutional-based cross-sectional study. PLoS One. 2021;16(2):e0247075.
6. Sitotaw B, Shiferaw W. Prevalence of Intestinal Parasitic Infections and Associated Risk Factors among the First-Cycle Primary Schoolchildren in Sasiga District, Southwest Ethiopia. J Parasitol Res. 2020;2020:8681247.
7. Mehraj V, Hatcher J, Akhtar S, Rafique G, Beg MA. Prevalence and factors associated with intestinal parasitic infection among children in an urban slum of Karachi. PLoS One. 2008;3(11):e3680.
8. Atabati H, Kassiri H, Shamloo E, Akbari M, Atamaleki A, Sahlabadi F, et al. The association between the lack of safe drinking water and sanitation facilities with intestinal Entamoeba spp infection risk: A systematic review and meta-analysis. PLoS One. 2020;15(11):e0237102.
9. Jemaneh L. The epidemiology of schistosomiasis mansoni and soil-transmitted helminths in elementary school children from the South Gondar Zone of the Amhara National Regional State, Ethiopia. Ethiopian Med J. 2000;38(2):105-18.
10. Tefera E, Mohammed J, Mitiku H. Intestinal helminthic infections among elementary students of Babile town, eastern Ethiopia. Pan African Med J. 2015;20:50.
11. Kloos H, Zein ZA. The ecology of health and disease in Ethiopia: Routledge; 2019.
12. Kumie A, Ali A. An overview of environmental health status in Ethiopia with particular emphasis to its organization, drinking water and sanitation: a literature survey. Ethiopian J Heal Dev. 2005;19(2):89.
13. Zenu S, Alemayehu E, Woldemichael K. Prevalence of intestinal parasitic infections and associated factors among street children in Jimma town; south West Ethiopia in 2019: a cross sectional study. BMC Pub Heal. 2019;19(1):1731.
14. Hailu GG, Ayele ET. Assessment of the prevalence of intestinal parasitic infections and associated habit and culture-related risk factors among primary schoolchildren in Debre Berhan town, Northeast Ethiopia. BMC Public Heal. 2021;21(1):112.
15. Asfaw ST, Giotom L. Malnutrition and enteric parasitoses among under-five children in Aynalem Village, Tigray. Ethiopian J Heal Dev. 2000;14(1):67-75.
16. Nyantekyi LA, Legesse M, Belay M, Tadesse K, Manaye K, Macias C, et al. Intestinal parasitic infections among under-five children and maternal awareness about the infections in Shesha Kekele, Wondo Genet, Southern Ethiopia. Ethiopian J Heal Dev. 2010;24(3).
17. Birrie H, Balcha F, Abebe F. Intestinal parasitoses among under-fives in two communities in Ethiopia. Ethiopian J Heal Dev. 1998;12(1).
18. Gelaw A, Anagaw B, Nigussie B, Silesh B, Yirga A, Alem M, et al. Prevalence of intestinal parasitic infections and risk factors among schoolchildren at the University of Gondar Community School, Northwest Ethiopia: a cross-sectional study. BMC Pub Heal. 2013;13:1-7.
19. Teklemariam A, Dejenie T, Tomass Z. Infection prevalence of intestinal helminths and associated risk factors among schoolchildren in selected kebeles of Enderta district, Tigray, Northern Ethiopia. J Parasitol Vector Biol. 2014;6(11):166-73.
20. Wale M, Wale M, Fekensa T. The prevalence of intestinal helminthic infections and associated risk factors among school children in Lumame town, Northwest, Ethiopia. J Parasitol Vector Biol. 2014;6(10):156-65.
21. Central Statistics Agency. 2013. http://www.statsethiopia.gov.et/wp-content/uploads/2019/05/ICPS-Population-Projection-2007-2037-produced-in-2012.pdf Population Projections for Ethiopia 2007-2037. Accessed 11 November, 2021.
22. Naing L, Winn T, Rusil BN. Practical issues in calculating sample size for prevalence studies. 2006; AOS **1**: 9–14
23. Williams J. District Laboratory Practice in Tropical Countries. Part 1. Monica Cheesbrough. Doddington. Cambridgeshire: Tropical Health Technology, 1998. :
24. Gebreslassie M, Dejenie T, Tomass Z. Prevalence of intestinal parasites and associated risk factors in school children of Aksum Town, Northern Ethiopia. Acta Parasitol Glob. 2015;6(1):42-8.
25. Tulu B, Taye S, Amsalu E. Prevalence and its associated risk factors of intestinal parasitic infections among Yadot primary school children of South Eastern Ethiopia: a cross-sectional study. BMC Res Notes. 2014;7(1):1-7.
26. Feleke DG, Alemu Y, Bisetegn H, Mekonnen M, Yemanebrhane N. Intestinal parasitic infections and associated factors among street dwellers and prison inmates: A systematic review and meta-analysis. PLoS One. 2021;16(8):e0255641.
27. Tigabu A, Taye S, Aynalem M, Adane K. Prevalence and associated factors of intestinal parasitic infections among patients attending Shahura Health Center, Northwest Ethiopia. BMC Res Notes. 2019;12(1):333.
28. Menjetta T, Simion T, Anjulo W, Ayele K, Haile M Tafesse T, et al. Prevalence of intestinal parasitic infections in Hawassa University students’ clinic, Southern Ethiopia: a 10-year retrospective study.. BMC Res Notes. 2019;12:702.
29. World Health Organization. *Guidelines for Drinking-Water Quality, V.2: Health Criteria and Other Supporting Information*. International Programme on Chemical Safety: Library Cataloguing in Publication Data. 2nd ed. Mastercom/Wiener Verlag; Austria, 1996; pp.: 15, 51.
30. Ministry of Water, Irrigation and Energy. 2019. [https://washdata.org/sites/default/files/documents/reports/2019-06/](https://washdata.org/sites/default/files/documents/reports/2019-06/Ethiopia_WASH_Sector_Statistical_Report_2018.pdf). National Hygiene and Sanitation Strategy (2019-2030). Accessed May 2, 2023.
31. United Nations Development Programme. 2020. https://www.et.undp.org/content/ethiopia/en/home/ourwork/environmentandenergy/focus-areas/water-sanitation-and-hygiene.html. Water, Sanitation and Hygiene. Accessed May 2, 2023.
32. Demographic Health survey. 2016. https://dhsprogram.com/pubs/pdf/FR328/FR328.pdf .key indicators report. Addis Ababa, Ethiopia, and Rockville, Maryland, USA Ethiopia: Ethiopia Demographic and Health Survey.
33. World Bank Group Ethiopia. 2018. . <https://projects.worldbank.org/en/projects-operations/project-detail/P146732> - Second Urban Water Supply and Sanitation Project. Accessed May 2, 2023.
34. Beyene A, Hailu T, Faris K, Kloos H. Current state and trends of access to sanitation in Ethiopia and the need to revise indicators to monitor progress in the Post-2015 era. BMC Pub Heal. 2015;15(1):451.
35. United Nations Children’s Fund (UNICEF). 2018. <https://www.unicef.org/ethiopia/media/478/file/WASH-in-Schools-in-Ethiopia-Policy-and-Implementation-Brief-2018.pdf>.WASH in Schools in Ethiopia: Policy and Implementation Brief. Accessed May 2, 2023.
36. USAID. 2018. <https://www.usaid.gov/ethiopia/water-sanitation-and-hygiene-wash-program>.Ethiopia Water, Sanitation, and Hygiene (WASH) Program. Accessed May 2, 2023.
37. World Health Organization. 2011. https://apps.who.int/iris/handle/10665/44671 Helminth control in school-age children: a guide for managers of control programmes. Accessed 12 Apr 2020.
38. World Health Organization. 2012. https://apps.who.int/iris/handle/10665/44804 Soil-transmitted helminthiasis, eliminating soil transmitted helminthiases as a public health problem in children, progress report 2001-2010 and strategic plan 2011-2020. Accessed 19 May 2020.
39. World Health Organization. 2015. https://apps.who.int/iris/handle/10665/152781 .Investing to overcome the global impact of neglected tropical diseases: third WHO report on neglected tropical diseases. Accessed 7 Jan 2021.
40. Osman M, El Safadi D, Cian A, Benamrouz S, Nourrisson C, Poirier P, et al. Prevalence and Risk Factors for Intestinal Protozoan Infections with Cryptosporidium, Giardia, Blastocystis and Dientamoeba among Schoolchildren in Tripoli, Lebanon. PLoS Neg Trop Dis. 2016;10(3):e0004496.
41. Zemene T, Shiferaw MB. Prevalence of intestinal parasitic infections in children under the age of 5 years attending the Debre Birhan referral hospital, North Shoa, Ethiopia. BMC Res Notes. 2018;11(1):58.
42. Liao CW, Fu CJ, Kao CY, Lee YL, Chen PC, Chuang TW, et al. Prevalence of intestinal parasitic infections among school children in capital areas of the Democratic Republic of São Tomé and Príncipe, West Africa. Afr Health Sci. 2016;16(3):690-7.
43. Adenowo AF, Oyinloye BE, Ogunyinka BI, Kappo AP. Impact of human schistosomiasis in sub-Saharan Africa. The Brazilian journal of infectious diseases : an official publication of the Brazilian Soci Infect Dis. 2015;19(2):196-205.
44. Ngui R, Ishak S, Chuen CS, Mahmud R, Lim YA. Prevalence and risk factors of intestinal parasitism in rural and remote West Malaysia. PLoS Neg Trop Dis. 2011;5(3):e974.
45. Quihui L, Valencia ME, Crompton DW, Phillips S, Hagan P, Morales G, et al. Role of the employment status and education of mothers in the prevalence of intestinal parasitic infections in Mexican rural schoolchildren. BMC Pub Heal. 2006;6:225.
46. El-Masry H, Ahmed Y, Hassan A, Zaky S, Abd-Allah E, El-Moselhy E, et al. Prevalence, risk factors and impacts of schistosomal and intestinal parasitic infections among rural school children in Sohag Governorate. Egyptian J Hosp Med. 2007;29(1):616-30.
47. Alula GA, Munshea A, Nibret E. Prevalence of Intestinal Parasitic Infections and Associated Risk Factors among Pregnant Women Attending Prenatal Care in the Northwestern Ethiopia. BioMed Res Inter. 2021;2021:3387742.
48. Woinishet A, Binyam G, Tewoderos S, Zelalem Negash M, Zemichael G. Prevalence of intestinal parasites and associated factors among food handlers in food establishments in the Lideta subcity of Addis Ababa, Ethiopia: an institution-based, cross-sectional study. BMJ open. 2022;12(7):e061688.
49. Marami D, Hailu K, Tolera M. Prevalence and associated factors of intestinal parasitic infections among asymptomatic food handlers working at Haramaya University cafeterias, eastern Ethiopia. Ann Occup Environ Med. 2018;30(1).
50. de Silva NR, Brooker S, Hotez PJ, Montresor A, Engels D, Savioli L. Soil-transmitted helminth infections: updating the global picture. Trend Parasitol. 2003;19(12):547-51.
51. Woldu W, Bitew BD, Gizaw Z. Socioeconomic factors associated with diarrheal diseases among under-five children of the nomadic population in northeast Ethiopia. Trop Med Heal. 2016;44:40.
52. Rahman A. Assessing income-wise household environmental conditions and disease profile in urban areas: study of an Indian city. Geo J. 2006;65:211-27.
53. Gezehegn D, Abay M, Tetemke D, Zelalem H, Teklay H, Baraki Z, et al. Prevalence and factors associated with intestinal parasites among food handlers of food and drinking establishments in Aksum Town, Northern Ethiopia. BMC Pub Heal. 2017;17(1):819.
54. Yeshanew S, Tadege M, Abamecha A. Prevalence and Associated Factors of Intestinal Parasitic Infections among Food Handlers in Mettu Town, Southwest Ethiopia. J Trop Med. 2021;2021:6669734.
55. Rop DC, Nyanchongi BO, Nyangeri J, Orucho VO. Risk factors associated with intestinal parasitic infections among inmates of Kisii prison, Kisii county, Kenya. BMC Res Notes. 2016;9(1):384.
56. Gupta R, Rayamajhee B, Sherchan SP, Rai G, Mukhiya RK, Khanal B, et al. Prevalence of intestinal parasitosis and associated risk factors among school children of Saptari district, Nepal: a cross-sectional study. Trop Med Heal. 2020;48:73.
57. Suen LKP, So ZYY, Yeung SKW, Lo KYK, Lam SC. Epidemiological investigation on hand hygiene knowledge and behaviour: a cross-sectional study on gender disparity. BMC Pub Heal. 2019;19(1):401.
58. Yusof AM, Mohammad M, Abdullahi MA, Mohamed Z, Zakaria R, Wahab RA. Occurrence of Intestinal Parasitic Contamination in Select Consumed Local Raw Vegetables and Fruits in Kuantan, Pahang. Trop Life Sci Res. 2017; 28(1):23-32.
