# Supplementary material for: Prevalence of intestinal parasitic infections and associated risk factors among patients attending Debarq Primary Hospital, northwest Ethiopia
Source: PLoS One. 2024 Mar 7;19(3):e0298767. doi: 10.1371/journal.pone.0298767 (PMC10919636; doi:10.1371/journal.pone.0298767)
Supplement: Attachment — Submitted filename: Response to Reviewers.docx [file pone.0298767.s004.docx]

Prevalence of human intestinal parasitic infections and associated risk factors in Debarq, northwest Ethiopia

Reviewer #2

Dear Reviewer,

Thank you for your suggestions and comments. Please find a point-by-point response to your comments and suggestions below.

1. Abstract- Line 30; how where the study participants recruited

- As suggested, the recruitment process is described in the abstract and methods sections.

2. **Introduction**
3. In paragraph one please add the African burden of IIPs

- The African burden of IPIs is described as suggested.

4. Line 55- was should be replaced with have

- Corrected as suggested.

5. Line 57- T.trichiura is appearing for the first time and should be written in full.

- Corrected as suggested.

6. Line 61-63 is a repletion of line 50-52 and should be deleted

- Lines 50-52 are about factors for IPIs in developing nations, and lines 61-63 are about factors for IPIs in Ethiopia. Because Ethiopia is a developing country, it shares factors with other developing countries.

7. Line 68-69 that statement is not true

- Corrected as suggested.

8. The introduction can be improved, it is information about the local context what about elsewhere? What are the current WHO recommendations about prevention and control of the parasites?

- Information on IPIs from elsewhere is included, as well as information on parasite prevention and control.

9. **Methods**
10. Sub -heading inclusion criteria is not needed in manuscript writing- can sampling procedure/recruitment of study participants

- The subheading is replaced with recruitment of study participants

11. Sub heading 100- can be replace with questionnaire survey

- The sub-heading is replaces with questionnaire survey as suggested.

12. Line 101; how where the study participants recruited?

- The recruitment process is described as suggested.

13. **Results**
14. Table three can be replaced with table 4

- Tables 3 and 4 were merged.

15. **Discussion**
16. Line 212- what about other countries with similar problems; what is their prevalence level?

- The prevalence of IPIs in other countries is described as suggested.

17. The discussion is very scanty- most of the associated risk factors have not been discussed and a reason given why they were associated and what others have found with similar studies.

- All of the associated factors are explored in light of your suggestions. The entire portion of discussion is revised.

18. The discussion needs a lot of revision- all the associated risk factors needs to be discussed.

- All of the associated factors are explored in light of your suggestions. The entire portion of discussion is revised.

19. Addition of qualitative data would have given more insight as to why this IIPs are still a problem.

- Because the study is quantitative and lacks qualitative data, we are unable to include the data you proposed in the revised version of the manuscript.

Reviewer #1

Dear Reviewer,

Thank you for your suggestions and comments. Please find a point-by-point response to your comments and suggestions below.

1) If the some results demonstrate as charts or graphs, the results could have more visibility, then you can your table as supplementary materials.

- Because of the nature of the data, including its being with many variables, we assumed that placing it in a table rather than charts and graphs would describe it more clearly and concisely.
