# Supplementary material for: Prevalence of intestinal parasitic infections and associated risk factors among patients attending Debarq Primary Hospital, northwest Ethiopia
Source: PLoS One. 2024 Mar 7;19(3):e0298767. doi: 10.1371/journal.pone.0298767 (PMC10919636; doi:10.1371/journal.pone.0298767)
Supplement: Attachment — Submitted filename: Plos One comments.docx [file pone.0298767.s005.docx]

**Prevalence of intestinal parasitic infections and associated 2 risk factors among patients attending Debarq Primary 3 Hospital, northwest Ethiopia**

1. Stool sample collection- Line 113-t0 155 can be rephrased. Also, in line 144, use past tense instead of present tense.
2. The conclusion can be improved by a more detailed recommendation. Health education can be changed to health promotion.
