# Supplementary material for: Prevalence of intestinal parasitic infections and associated risk factors among patients attending Debarq Primary Hospital, northwest Ethiopia
Source: PLoS One. 2024 Mar 7;19(3):e0298767. doi: 10.1371/journal.pone.0298767 (PMC10919636; doi:10.1371/journal.pone.0298767)
Supplement: Attachment — Submitted filename: Review comments for PONE-D-23-15672.docx [file pone.0298767.s006.docx]

**Review comments for PONE -D-23-15672 manuscript**

**Manuscript title:** Prevalence of human intestinal parasitic infections and associated risk factors in Debarq, northwest Ethiopia

**General comments on the manuscript**

The manuscript talks about the Prevalence of human intestinal parasitic infections and associated risk factors in Debarq, northwest Ethiopia. It brings out very useful information of public health concern. More often than not, people in communities do harbour many intestinal parasitic infections which are not noticed by individuals affected. Only when these cause a problem, do such individuals seek medical attention. It is interesting to note the extent of these intestinal infections in individuals who sort medical attention for different reasons other than those related to intestinal parasitic infections was quite high. Actually, the results indicate that with good or improved sanitation practices, most of these and similar infection could be prevented. It also showed that, the older the individual, the more the likelihood of having multiple IPIs. That said, the manuscript is generally well written but is without grammatical errors. Below are my comments.

**Specific comments on the manuscript.**

**Abstract:** NONE

**Introduction:**

Line 57. Put space between T and full stop to read as *T. trichiura*

**Materials and methods:**

**Study area and population:**

Line 80: Leave a space between longitude.The

**Inclusion and exclusion criteria:**

Each of these criteria to have its own paragraph.

**Stool examination:**

**Direct wet / iodine mount:**

Line 122: replace “was” with “were” …………, in approximately 2 grams of stool was emulsified……………

Line 127: delete comma after eggs in ………………… cysts, eggs, and trophozoites of motile intestinal parasites.

**Formal-ether concentration:**

Line 137: Delete comma after eggs in ………………if helminth eggs, larvae, cysts, and/or protozoan trophozoites

***Question:*** *Why did the authors use two methods of stool analysis without showing the results for each of the methods used? I do not see the need of including analysis whose results you do not use. Unless, they present the results for each of the methods used, they need to only pick one method*.

**Results:**

**Characteristics of the study participants:**

Lines 150 and 151: Add “d” on the two words of “live”) ……………While 57.35% of participants live in urban areas, 42.65% live in rural areas. The results have to be stated in a reported speech.

**Prevalence of intestinal parasitic infections:**

Line 162: Add “mostly” between “and “and “in”……in participants aged 25 and older and in men (17.06%).

**Discussion:**

Lines 218 to 219: Capitalise each word of the…………… world health organization (WHO) to read as ………….World Health Organization”…………..

Line 221: Replace “norm” with “normal” ………………resistant to the norm concentration of chlorine in drinking water

Line 229: …………. area, such issues must be identified and addressed. Please note that this study has already identified them and I guess what is needed now is to address them. Thus delete: “must be identified and”

Line 233: Add “were” between residents and engaged in ……………….. and 37.5% of rural residents engaged in open defecation,

Line 237: See previous comment made on Line 229. Please delete “identify and” in ………………. it is essential to identify and address sanitary facility concerns in…………..

Line 248: Replace “are” with “is” in …………….. launching similar programs are likely to reduce the prevalence of intestinal………… because this sentence is talking about the implementation of the WASH programme.

Line 259: Add “be” between to and associate in …………..has also been found to associate with an increased risk of intestinal………….. Further, replace “associate” with “associated”

Line 273: Replace “are” with “being” in ……………….with protozoan parasites (E. histolytica and G. lamblia) are predominantly…………

Line 274: Delete “a” on “abe” in ……………… of intestinal parasites was observed to abe associated…………………….
